# The modified Blumgart anastomosis is associated with a lower incidence of clinically relevant postoperative pancreatic fistula and a shorter anastomotic time: a prospective cohort study with a historical control group

**DOI:** 10.1016/j.sipas.2026.100360

**Published:** 2026-09-18

**Authors:** Hideaki Sato, Shuichi Aoki, Ryosuke Kashiwagi, Mika Ando, Yuichiro Umino, Mitsuhiro Shimura, Koetsu Inoue, Masahiro Iseki, Daisuke Douchi, Takayuki Miura, Shimpei Maeda, Masaharu Ishida, Masamichi Mizuma, Michiaki Unno

**Affiliations:** Department of Surgery, Tohoku University Graduate School of Medicine, Sendai, Japan

**Keywords:** Blumgart anastomosis, Pancreatic fistula, Pancreaticoduodenectomy, Pancreaticojejunostomy

## Abstract

**Introduction:**

The modified Blumgart anastomosis (mBA) is widely used for pancreaticojejunostomy; however, its clinical benefits remain uncertain.

**Methods:**

We prospectively enrolled 179 consecutive patients scheduled to undergo pancreaticoduodenectomy (PD) at our institution between February 2016 and December 2018; 125 underwent PD with mBA and were included in the analysis. Short-term outcomes were compared with those of a historical cohort of 103 patients who underwent conventional anastomosis (CA) between April 2012 and January 2016.

**Results:**

Baseline demographic characteristics did not differ significantly between the two groups. The incidences of clinically relevant postoperative pancreatic fistula (POPF) and grade B/C postpancreatectomy hemorrhage (PPH) were significantly lower in the mBA group than in the CA group (7.2%vs. 24.3%, p < 0.001; 4.0% vs. 11.7%, p = 0.028, respectively). The anastomotic time was also significantly shorter in the mBA group (p < 0.001), whereas the overall operative time did not differ significantly (p = 0.206). Multivariable analysis showed that mBA was independently associated with lower odds of clinically relevant POPF, and the incidence remained significantly lower after propensity score matching (PSM).

**Discussion:**

The mBA technique may reduce shear stress on the pancreatic parenchyma, potentially contributing to the lower incidences of POPF and PPH.

**Conclusions:**

The mBA was associated with lower incidences of clinically relevant POPF and PPH and with a shorter anastomotic time, suggesting potential clinical advantages of this technique.

## Highlights

• The modified Blumgart anastomosis (mBA) was associated with a lower POPF rate.
• mBA was associated with a shorter anastomotic time.
• The incidence of postpancreatectomy hemorrhage was lower in the mBA group.
• Lower POPF rates with mBA persisted after propensity score matching.
• The simplicity of mBA may facilitate surgical standardization.

## 1 Introduction

Pancreaticoduodenectomy (PD) is the standard surgical procedure for neoplasms of the pancreatic head and periampullary region, including tumors of the distal bile duct, duodenum, and ampulla of Vater [[1], [2], [3]]. The procedure was first reported by Whipple in 1935, with an operative mortality rate of 25% in the initial series [1]. PD is a complex procedure associated with substantial postoperative morbidity [4]. Advances in surgical techniques, perioperative care, and centralization have improved its safety [5,6], and recent reports indicate mortality rates below 3% at high-volume centers [7,8]. Nevertheless, PD remains associated with a substantial risk of postoperative complications, including postoperative pancreatic fistula (POPF), hemorrhage, and POPF-related intra-abdominal abscess [9,10].

The reported incidence of POPF after pancreaticojejunostomy (PJ) ranges from 9.9% to 28.5% according to the definitions of the International Study Group of Pancreatic Surgery (ISGPS) [[11], [12], [13]]. Clinically relevant POPF often requires intensive postoperative management and prolongs hospitalization. Established risk factors include a soft pancreatic texture, a small main pancreatic duct (MPD; < 3 mm), and substantial intraoperative blood loss (> 500 mL) [14,15]. A high body mass index (BMI), advanced age, and male sex may also increase the risk of POPF [14,16].

PJ is a technically demanding component of PD [17], and various techniques have been developed to reduce the incidence of POPF [[18], [19], [20], [21], [22]]. Blumgart and colleagues described a duct-to-mucosa PJ reinforced with transpancreatic U-shaped mattress sutures [20,21]. Fujii et al. subsequently simplified this technique, creating what is now known as the modified Blumgart anastomosis (mBA) [22]. Recent studies have reported favorable outcomes with mBA, including a lower incidence of POPF [17,[22], [23], [24], [25], [26], [27], [28]].

At our institution, we introduced mBA in February 2016 and prospectively collected outcomes thereafter. In this study, we evaluated the surgical outcomes of mBA by comparing them with those of CA, the technique used in nearly all patients undergoing PD at our institution through January 2016.

## 2 Methods

### 2.1 Ethics

This study was conducted in accordance with the Declaration of Helsinki and was approved by the Institutional Review Board of Tohoku University on January 25, 2016 (approval no. 2015-1-689). The study was registered in the UMIN Clinical Trials Registry (UMIN000020813).

### 2.2 Patients and study outline

Patients in the mBA group were prospectively enrolled between February 2016 and December 2018. Initially, 179 consecutive patients scheduled to undergo PD were assessed for eligibility; 125 patients who underwent PD with mBA were included in the final analysis. The remaining 54 patients were excluded because the planned resection was not performed, they underwent total pancreatectomy or minimally invasive surgery (MIS), a non-mBA technique was used, or data were insufficient. MIS cases were excluded to maintain comparability with the historical cohort because MIS had not been widely adopted as a standard approach at our institution through 2015. The mBA data were collected prospectively, whereas the historical control group comprised 103 patients who underwent CA between April 2012 and January 2016. Thus, 228 patients who underwent PD at Tohoku University Hospital between April 2012 and December 2018 were included (Fig. 1). Sex was recorded as male or female according to the medical records. Morbidity was defined as Clavien-Dindo grade IIIa or higher [29]. POPF was defined and graded according to the 2016 ISGPS criteria [30], and postpancreatectomy hemorrhage (PPH) was defined and graded according to the ISGPS criteria [31]. Mortality was defined as in-hospital death. To account for baseline pancreatic fistula risk, the Fistula Risk Score (FRS) was calculated for each patient from four intraoperative variables: pancreatic texture, MPD diameter, pathology, and blood loss [32]. This study was reported in accordance with the Strengthening the Reporting of Observational Studies in Epidemiology (STROBE) statement.

**Fig. 1:**
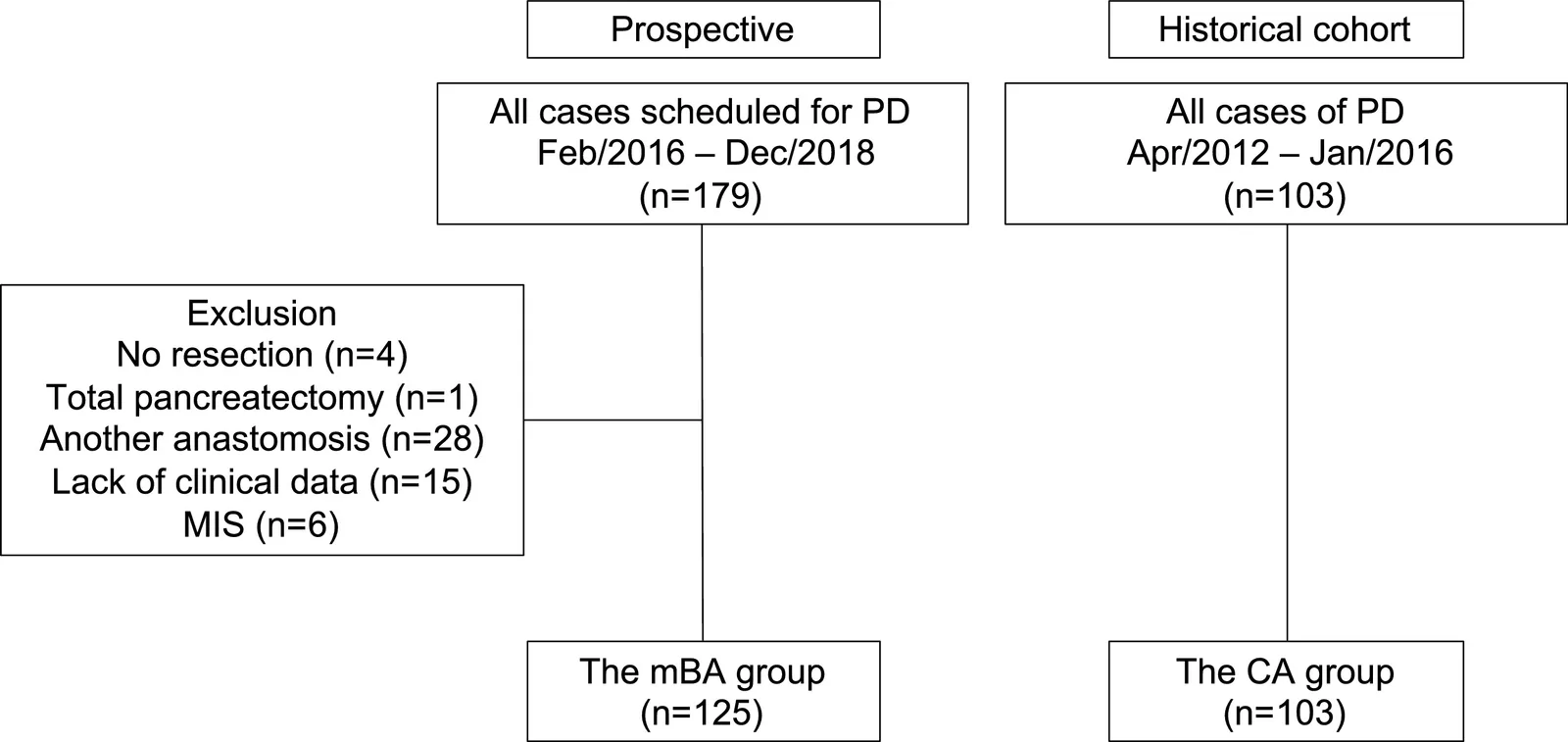
Case selection flowchart.

### 2.3 Surgical technique

At our institution, subtotal stomach-preserving pancreaticoduodenectomy (SSPPD) was the standard form of PD. Lymph node dissection was performed as indicated and included nodes in the hepatoduodenal ligament, around the celiac axis and superior mesenteric artery, and around the pancreatic head. The pancreatic parenchyma was transected using electrocautery or an ultrasonic coagulating and cutting device. For reconstruction, the jejunum was brought up through a retrocolic route, and the modified Child method was used in all cases. An external pancreatic duct stent was generally preferred; however, the decision to place a stent was based on intraoperative findings, including pancreatic texture and MPD diameter, and the stent could be omitted in selected cases. Internal pancreatic stents were not used during the study period. All procedures were performed by dedicated gastrointestinal and hepatobiliary-pancreatic (HBP) surgical teams. To maintain technical consistency across the two periods, the primary surgeons were either board-certified HBP surgeons with 10–25 years of experience or senior residents. When a senior resident served as the primary surgeon, a board-certified HBP surgeon served as the first assistant and provided direct supervision.

### 2.4 Conventional anastomosis

The CA technique consisted of a two-layer, end-to-side anastomosis between the pancreas and jejunum. A small jejunotomy was created on the antimesenteric side of the jejunum. First, the posterior pancreatic parenchyma was sutured to the jejunum using interrupted 5–0 nonabsorbable monofilament sutures. The pancreatic duct was then anastomosed circumferentially to the jejunal mucosa using eight interrupted 6–0 absorbable monofilament sutures. Finally, the anterior pancreatic parenchyma was sutured to the jejunum using interrupted 5–0 nonabsorbable monofilament sutures. An external pancreatic duct stent was placed when the MPD diameter was ≤ 3 mm and omitted when it was > 3 mm [33].

### 2.5 Modified Blumgart anastomosis

The mBA was an end-to-side PJ originally described by Blumgart [20] and subsequently modified by Fujii [22]. A small jejunotomy was created on the antimesenteric side of the jejunum. Six transpancreatic jejunal seromuscular stitches were placed using three double-armed 3-0 nonabsorbable monofilament sutures. A duct-to-mucosa anastomosis was then constructed circumferentially using eight interrupted 6-0 absorbable monofilament sutures, as in the CA technique. After completion of the duct-to-mucosa anastomosis, the anterior jejunal wall was secured to the anterior surface of the pancreatic remnant using the same three double-armed 3-0 nonabsorbable monofilament sutures in a mattress fashion. An external pancreatic duct stent was routinely placed.

### 2.6 Statistical analysis

All statistical analyses were performed using JMP® Student Edition 18.2.2 for Macintosh (SAS Institute Inc., Cary, NC, USA). Continuous variables were compared using the Wilcoxon rank-sum test, and categorical variables were compared using Fisher's exact test or the chi-square test. Factors associated with clinically relevant POPF were examined using univariable and multivariable logistic regression analyses. PSM was used to adjust for potential clinical and intraoperative confounders. The propensity score model included patient characteristics (age, sex, BMI, diabetes mellitus [DM], and hypertension [HT]) and surgical factors (operative time, blood loss, MPD diameter, pancreatic texture, and pancreatic stent use). One-to-one nearest-neighbor matching was performed with a caliper width of 0.2. A p value < 0.05 was considered statistically significant.

## 3 Results

### 3.1 Characteristics of the patients

Patient characteristics are shown in Table 1. A total of 228 patients who underwent PD were included (125 in the mBA group and 103 in the CA group). The median age of the entire cohort was 68 years (range, 19–84 years), and 148 patients (64.9%) were male. Baseline clinicodemographic characteristics did not differ significantly between the groups, including sex, age, preoperative BMI, history of DM or HT, antithrombotic drug use, diagnosis, and receipt of neoadjuvant therapy. The mBA group comprised 85 male and 40 female patients, with a median age of 67 years (range, 19–84 years); the CA group comprised 63 male and 40 female patients, with a median age of 68 years (range, 19–82 years). Pancreatic cancer was the most common diagnosis in both groups, occurring in 53 (42.4%) and 49 patients (47.6%) in the mBA and CA groups, respectively.

**Table 1:** Demographics and clinical characteristics of 228 patients.

Group p	mBA	CA
Number (case)	125 (54.8%)	103 (45.2%)
Age (year) 0.482	67 (19–84)	68 (19–82)
Sex (male/female) 0.282	85 / 40	63 / 40
BMI (kg/m^2^) 0.161	22.6 (16.9–32.5)	21.9 (15.8–29.6)
DM (case) 0.910	44 (35.2%)	37 (35.9%)
HT (case) 0.317	69 (55.2%)	50 (48.5%)
Antithrombotic (case) 0.738	24 (19.2%)	18 (17.5%)
Disease (case)	0.681	
Pancreatic Cancer	53 (42.4%)	49 (47.6%)
IPMN or IPMC	18 (14.4%)	11 (10.7%)
Cholangiocarcinoma	24 (19.2%)	16 (15.5%)
Other	30 (24.0%)	27 (26.2%)
Neoadjuvant therapy 0.615	34 (27.2%)	25 (24.3%)

*Values are medians (range).

Abbreviations are BMI, body mass index; DM, diabetes mellitus; HT, hypertension; IPMN, intraductal papillary mucinous neoplasm; IPMC, intraductal papillary mucinous carcinoma.

### 3.2 Surgical outcomes

Surgical outcomes are summarized in Table 2. Operative time, blood loss, MPD diameter, pancreatic texture, FRS (p = 0.947), and postoperative anticoagulant therapy did not differ significantly between the groups. By contrast, the rates of combined vascular resection and pancreatic stent use and the anastomotic time differed significantly. In the mBA group, 27 patients (21.6%) underwent vascular resection and 124 (99.2%) received a pancreatic stent, compared with 37 (35.9%) and 55 (53.4%), respectively, in the CA group (p = 0.017 and p < 0.001, respectively). Despite the higher rate of vascular resection in the CA group, operative time and blood loss did not differ significantly. The anastomotic time was significantly shorter in the mBA group than in the CA group (median, 33 min [range, 13–55] vs. 44 min [range, 23–73]; p < 0.001) (Fig. 2).

**Table 2:** Surgical outcomes of 228 patients.

Group p	mBA (n = 125)	CA (n = 103)
Operative time (min) 0.206	516 (209–1345)	534 (295–1021)
Blood loss (mL) 0.275	1006 (100–4813)	1120 (150–9695)
Vascular resection (case) 0.017	27 (21.6%)	37 (35.9%)
MPD (mm) 0.749	4 (1–8)	4 (1–15)
Texture (soft / hard) 0.232	73 / 52	52 / 51
Pancreatic stent (case) < 0.001	124 (99.2%)	55 (53.4%)
Anticoagulants (case) 0.068	71 (56.8%)	46 (44.7%)
Anastomotic time (min) < 0.001	33 (13–55)	44 (23–73)
Fistula risk score 0.947	6 (1–9)	5 (1–10)

*Values are medians (range).

Abbreviations are MPD, main pancreatic duct.

**Fig. 2:**
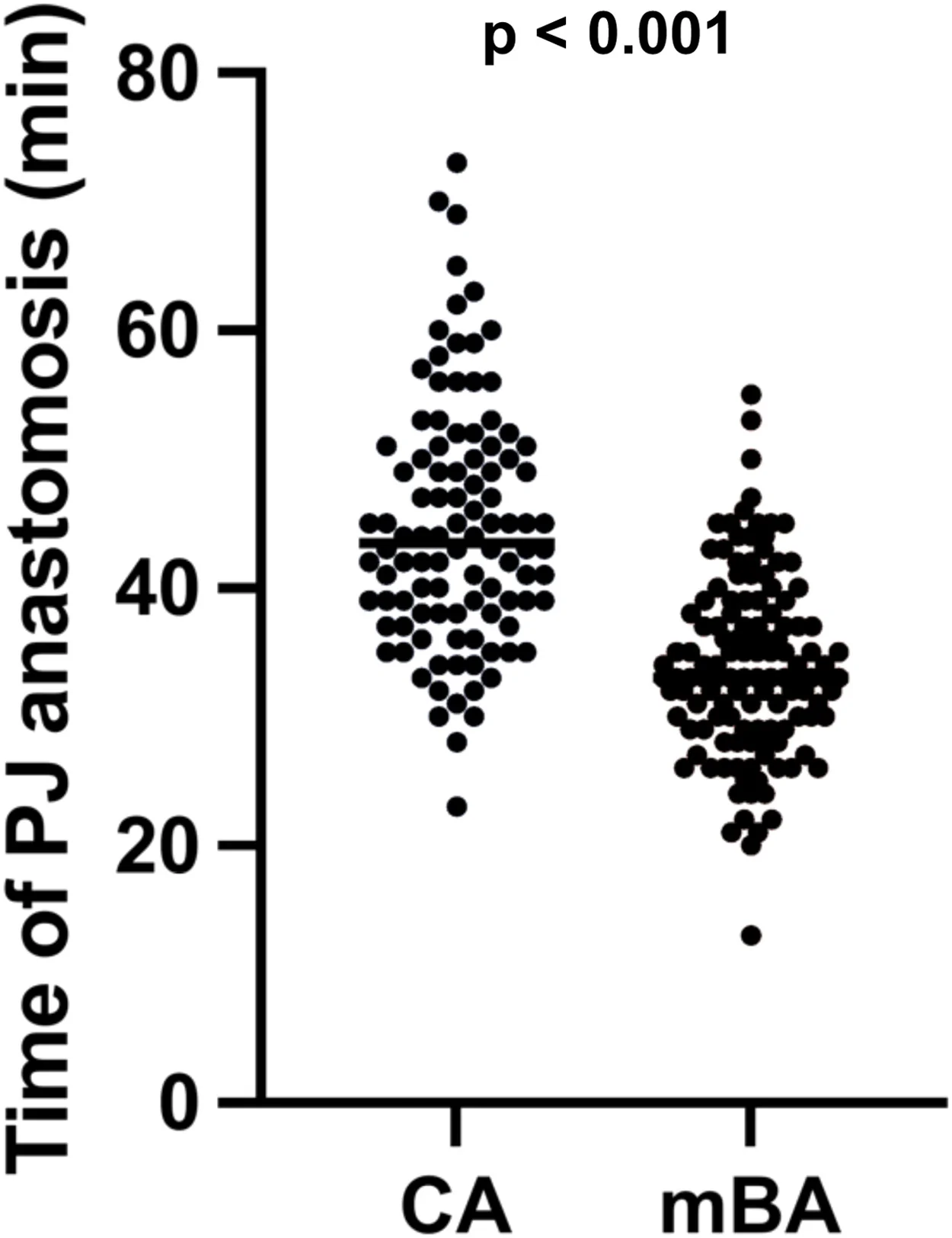
Comparison of anastomotic time between the mBA and CA groups. Anastomotic time was significantly shorter in the mBA group than in the CA group.

### 3.3 Postoperative outcomes

Postoperative outcomes are presented in Table 3. The rates of clinically relevant POPF and grade B/C PPH were significantly lower in the mBA group than in the CA group (7.2%vs. 24.3%, p < 0.001; 4.0% vs. 11.7%, p = 0.028, respectively). The rates of drain reinsertion and Clavien-Dindo grade III or higher morbidity were also significantly lower in the mBA group (4.0%vs. 14.6%, p = 0.005; 21.6% vs. 37.9%, p = 0.007, respectively). No significant differences were observed in biliary leakage, time to drain removal, or postoperative hospital stay. One patient in each group died during the hospital stay.

**Table 3:** Postoperative outcomes of 228 patients.

Group p	mBA (n = 125)	CA (n = 103)
C-D ≥ III (case) 0.007	27 (21.6%)	39 (37.9%)
DGE	8 (6.4%)	12 (11.7%)
Abdominal abscess	11 (8.8%)	20 (19.4%)
Reoperation	3 (2.4%)	4 (3.9%)
POPF (case) 0.097	61 (48.8%)	39 (37.9%)
None	64 (51.2%)	64 (62.1%)
BL	52 (41.6%)	14 (13.6%)
CR	9 (7.2%)	25 (24.3%)
< 0.001		
PPH grade B/C (case) 0.028	5 (4.0%)	12 (11.7%)
Biliary leakage (case) 0.336	4 (3.2%)	6 (5.8%)
Drain removal day (day) 0.212	6 (4–124)	6 (4–275)
Drain reinsertion (case) 0.005	5 (4.0%)	15 (14.6%)
Hospital stays (day) 0.140	31 (13–113)	34 (5–419)
Mortality (case) 0.891	1 (0.8%)	1 (1.0%)

*Values are medians (range).

Abbreviations are C-D, Clavien-Dindo classification; DGE, delayed gastric emptying; POPF, postoperative pancreatic fistula; BL, biochemical leak; CR, clinically relevant; PPH, postpancreatectomy hemorrhage.

### 3.4 Factors associated with clinically relevant POPF

Factors associated with clinically relevant POPF are shown in Table 4. Univariable analysis identified BMI < 25 kg/m² (odds ratio [OR], 0.355; p = 0.028), absence of DM (OR, 0.420; p = 0.041), mBA (OR, 0.242; p < 0.001), and pancreatic stent use (OR, 0.311; p = 0.034) as factors associated with lower odds of clinically relevant POPF. In multivariable analysis, BMI < 25 kg/m² (OR, 0.271; 95% confidence interval [CI], 0.10–0.76; p = 0.014), mBA (OR, 0.117; 95% CI, 0.04–0.27; p < 0.001), and pancreatic stent use (OR, 0.123; 95% CI, 0.03–0.41; p < 0.001) remained independently associated with lower odds of clinically relevant POPF.

**Table 4:** Univariable and multivariable analyses of factors associated with clinically relevant POPF.

Univariable analysis	Multivariable analysis				
	N	OR	p	OR	CI (95%) p
Male	148	0.989	0.978		
BMI < 25 kg/m^2^	197	0.355	0.028	0.271	[0.10, 0.76] 0.014
Pancreatic cancer	102	0.541	0.111		
Neoadjuvant therapy	59	0.570	0.219		
Non-DM	147	0.420	0.041	0.546	[0.19, 1.44] 0.227
Non-HT	109	0.965	0.925		
No use of antithrombotic	186	0.691	0.417		
mBA	125	0.242	<0.001	0.117	[0.04, 0.27] <0.001
Pancreatic stent	179	0.311	0.034	0.123	[0.03, 0.41] <0.001
Combined vascular resection	64	0.502	0.127		

Abbreviations are BMI, body mass index; DM, diabetes mellitus; HT, hypertension; mBA, modified Blumgart anastomosis.

### 3.5 Postoperative outcomes after PSM

Patient characteristics after PSM are shown in Supplementary Table 1, and postoperative outcomes are presented in Supplementary Table 2. PSM matched 47 of 125 patients in the mBA group with 47 of 103 patients in the CA group, forming the mBA-PSM and CA-PSM groups, respectively. After matching, clinicodemographic variables did not differ significantly between the groups, including age, sex, preoperative BMI, DM, HT, antithrombotic drug use, diagnosis, and receipt of neoadjuvant therapy. No significant between-group differences were observed in surgical factors, including MPD diameter, pancreatic texture, pancreatic stent use, and postoperative anticoagulant therapy (Supplementary Table 1). The rates of clinically relevant POPF and grade B/C PPH were significantly lower in the mBA-PSM group than in the CA-PSM group (8.5%vs. 42.6%, p < 0.001; 2.1% vs. 19.1%, p = 0.004, respectively). The rates of Clavien-Dindo grade III or higher morbidity and drain reinsertion were also significantly lower in the mBA-PSM group (25.5%vs. 51.1%, p = 0.010; 4.3% vs. 27.7%, p = 0.002, respectively).

## 4 Discussion

Several studies have reported favorable surgical outcomes with mBA after PD [[22], [23], [24], [25], [26], [27]], including less intraoperative blood loss, shorter operative time, a lower incidence of POPF, and shorter hospitalization [17,[22], [23], [24], [25], [26]]. In the present study, although overall morbidity and postoperative hospital stay did not differ significantly between the groups, the rates of clinically relevant POPF, grade B/C PPH, and drain reinsertion were significantly lower and the anastomotic time was significantly shorter in the mBA group than in the CA group. Unlike most previous reports, which were retrospective observational studies [[24], [25], [26], [27], [28]], data for the mBA cohort in the present study were collected prospectively.

The mBA technique may reduce shear stress on the fragile pancreatic parenchyma by using transpancreatic U-shaped mattress sutures to approximate the jejunal seromuscular layer to the pancreatic remnant during knot tying [20]. Leakage at the PJ site may result from tangential tension and shear forces on the pancreatic parenchyma, potentially disrupting the pancreatic capsule or tissue at the suture or knot sites. In high-risk patients with a soft pancreas and a small MPD, anastomotic integrity may be compromised by geometric mismatch and tissue fragility [34]. Among the available PJ techniques, mBA may therefore offer a technical advantage by limiting these stresses. In the present study, mBA was associated with a significantly lower incidence of clinically relevant POPF.

Although previous studies have reported shorter overall operative times with mBA [[24], [25], [26]], these findings may also reflect broader advances in surgical techniques and devices. Differences in total operative time therefore cannot be attributed to the anastomotic method alone. In our cohort, however, mBA was associated with a significantly shorter anastomotic time. A simpler and faster anastomosis may facilitate procedural standardization and surgical training.

In the CA group, a pancreatic duct stent was placed when the MPD diameter was ≤ 3 mm, whereas a stent was routinely placed in the mBA group. Consequently, pancreatic stent use was significantly more frequent in the mBA group. Pancreatic stenting, particularly in patients with a narrow MPD, has been reported to reduce the incidence of POPF [9,33]. After PSM, which included pancreatic stent use in the propensity score model, the incidence of clinically relevant POPF remained significantly lower in the mBA group. Nevertheless, because of the nonrandomized historical-control design, these findings demonstrate an association but do not establish an independent causal effect of mBA.

Although mBA was associated with a lower incidence of clinically relevant POPF, it was not associated with a shorter postoperative hospital stay. In Japan, the timing of postoperative discharge may be influenced by the healthcare system and patient preferences. In this cohort, postoperative hospital stay was approximately 30 days in both groups, and many patients were discharged after drain removal and recovery to their preoperative functional status. Thus, a lower incidence of POPF alone may not directly translate into a shorter hospital stay in this setting.

The incidence of PPH in this study was higher than that in previous reports. Fewer than half of the patients had pancreatic cancer, and more than half had a soft pancreas, indicating that the cohort included a high proportion of patients at risk of POPF and related hemorrhage. Nevertheless, only one in-hospital death occurred in each group, suggesting acceptable perioperative safety.

This study has several limitations. First, it was a single-center study comparing a prospectively collected mBA cohort from February 2016 to December 2018 with a historical CA cohort from April 2012 to January 2016. This nonrandomized historical comparison introduced potential temporal, selection, and perioperative-care biases. Although the standardized perioperative management protocol remained fundamentally consistent throughout the study period, advances in surgical proficiency, techniques, and devices may have favorably influenced outcomes in the more recent mBA group. Second, the sample size was relatively small, even though our institution is a high-volume pancreatic surgery center in Japan. Third, changes in primary surgeons between the two periods may have introduced surgeon-related confounding. Fourth, pancreatic stent use differed substantially between the groups, and multivariable analysis identified stent use as independently associated with lower odds of POPF. Although pancreatic stent use was included in the PSM model, residual confounding cannot be excluded, and the lower incidence of POPF cannot be attributed solely to the anastomotic technique. Finally, intraoperative blood loss was relatively high compared with recent benchmarks, likely reflecting the complexity of cases treated at our tertiary referral center, including those requiring extensive retroperitoneal dissection or major vascular reconstruction. A large, multicenter randomized controlled trial would be needed to address these limitations and evaluate the effects of mBA more definitively.

In conclusion, mBA was associated with lower incidences of clinically relevant POPF and PPH and with a shorter anastomotic time. Its technical simplicity may facilitate skill acquisition and procedural standardization, potentially improving surgical outcomes.

## Ethics statements

This study was conducted in accordance with the Declaration of Helsinki and was approved by the Institutional Review Board of Tohoku University on January 25, 2016 (approval no. 2015-1-689). Written informed consent for the use of clinical data was obtained from all patients.

## Declaration of generative AI and AI-assisted technologies in the manuscript preparation process

During the preparation of this work, the authors used ChatGPT and Gemini (Google) for language editing, translation, and text formatting. After using these tools, the authors reviewed and edited the content as needed and take full responsibility for the content of the published article.

## Funding

This research did not receive any specific grant from funding agencies in the public, commercial, or not-for-profit sectors.

## CRediT authorship contribution statement

**Hideaki Sato:** Writing – original draft, Investigation, Data curation, Conceptualization. **Shuichi Aoki:** Validation, Project administration, Formal analysis, Data curation, Conceptualization. **Ryosuke Kashiwagi:** Data curation. **Mika Ando:** Data curation. **Yuichiro Umino:** Data curation. **Mitsuhiro Shimura:** Data curation. **Koetsu Inoue:** Data curation. **Masahiro Iseki:** Data curation. **Daisuke Douchi:** Data curation. **Takayuki Miura:** Data curation. **Shimpei Maeda:** Data curation. **Masaharu Ishida:** Data curation. **Masamichi Mizuma:** Data curation. **Michiaki Unno:** Data curation.

## Declaration of competing interest

The authors declare that they have no known competing financial interests or personal relationships that could have appeared to influence the work reported in this paper.

## Data Availability

The datasets analyzed in this study are not publicly available because of hospital regulations governing patient privacy and institutional data protection. De-identified clinical data supporting the findings are available from the corresponding author upon reasonable academic request.
