# Supplementary material for: The modified Blumgart anastomosis is associated with a lower incidence of clinically relevant postoperative pancreatic fistula and a shorter anastomotic time: a prospective cohort study with a historical control group
Source: Surg Pract Sci. 2026 Sep 18;27:100360. doi: 10.1016/j.sipas.2026.100360 (PMC13628229; doi:10.1016/j.sipas.2026.100360)
Supplement: Supplementary file 1 [file mmc1.pdf]

Publication Checklist

Manuscript title: The modified Blumgart anastomosis reduces clinically relevant postoperative pancreatic fistula and shortens the anastomosis time: A prospective cohort study.

*For the Guide for Authors, the *Authorship & Conflicts of Interest Statement* form, and the *Copyright Transfer Agreement* form, please go to the Journal's website at http://www.e-asianjournalsurgery.com/

Only complete manuscript submissions will be considered for publication. Complete submission must include:

- [x] Cover letter for manuscript submission signed by the corresponding author
- [x] Authorship & conflicts of interest statement signed by all authors listed in the manuscript
- [x] Copyright transfer agreement signed by all authors listed in the manuscript
- [x] Manuscript in MS Word format

AND, where applicable

- [x] Letter of approval from review committee for use of human samples in research and human experiments
- [ ] Letter of approval from relevant authority for use of animals in experiments
- [ ] CONSORT flow chart for randomized controlled trial
- [ ] Signed consent to publish (in print and online) from human subjects who can be identified in your manuscript
- [ ] Letter(s) of permission from copyright holder(s) to use or adapt copyrighted sources in your manuscript

In the actual article, the following information must be provided:

- [x] Title page
  - [x] Article category
  - [x] Article title
  - [x] Name(s) and affiliation(s) of author(s)
  - [x] Conflicts of interest statement
  - [x] Corresponding author details (name, e-mail, mailing address, telephone and fax numbers)
  - [x] Running title not exceeding 50 characters
- [x] Abstract (no more than 250 words)
- [x] 3-5 keywords in alphabetical order for all article categories except Letters to the Editor (MeSH terms are recommended; see http://www.ncbi.nlm.nih.gov/mesh?term)
- [x] Main text
- [x] References in the correct format, cited in numerical order, and all references in the List are cited in the Text/Tables/Figures, and vice versa

AND, where applicable

- [x] Acknowledgments
- [x] Table headings and tables, each on a new page
- [x] Figure legends, on a new page
- [x] Electronic picture files of all figures; resolution of 300 dpi for halftone images, 600 dpi for combination art (halftone + line art), and 1000 dpi for line art

The following must be provided:

- [x] All authors acknowledge and agree that they will be charged a publication fee of $1200 USD upon acceptance of this article.

Corresponding author's name (typed)	Corresponding author's signature	Date
Shuichi Aoki	[signature]	Feb-10-2026

(on behalf of all the listed authors in the manuscript)
