# Supplementary material for: The modified Blumgart anastomosis is associated with a lower incidence of clinically relevant postoperative pancreatic fistula and a shorter anastomotic time: a prospective cohort study with a historical control group
Source: Surg Pract Sci. 2026 Sep 18;27:100360. doi: 10.1016/j.sipas.2026.100360 (PMC13628229; doi:10.1016/j.sipas.2026.100360)
Supplement: Supplementary file 2 [file mmc2.docx]

**Supplementary Table 1. Demographics and Clinical Characteristics of 94 patients after PSM**

**Group	mBA-PSM	CA-PSM	p**

**Number (case)	47 (50.0%)	47 (50.0%)**

**Age (year)	66 (19-83)	67 (19-79)	0.292**

**Sex (male / female)	28 / 19	30 / 17	0.671**

**BMI (kg/m^2^)	22.6 (17.3-32.5)	22.3 (15.8-29.6)	0.309**

**DM (case)	9 (19.2%)	10 (21.3%)	0.797**

**HT (case)	23 (48.9%)	24 (51.1%)	0.837**

**Antithrombotic (case)	7 (14.9%)	7 (14.9%)	1.000**

**Disease (case)	0.723**

**Pancreatic Cancer	10 (21.3%)	12 (25.5%)**

**IPMN or IPMC	5 (10.6%)	3 (6.4%)**

**Cholangiocarcinoma	23 (48.9%)	20 (42.6%)**

**Other	9 (19.2%)	12 (25.5%)**

**Neoadjuvant therapy	5 (10.6%)	6 (12.8%)	0.748**

**Vascular resection (case)	8 (17.0%)	9 (19.1%)	0.789**

**MPD (mm)	2 (1-7)	2 (1-6)	0.415**

**Texture (soft / hard)	39 / 8	38 / 9	0.789**

**Pancreatic stent (case)	46 (97.9%)	47 (100.0%)	0.237**

**Anticoagulants (case)	26 (55.3%)	19 (40.4%)	0.148**

***Values are medians (range)**

**Abbreviations are PSM, propensity score matching; BMI, body mass index; DM, diabetes mellitus; HT, hypertension; IPMN, intraductal papillary mucinous neoplasm; IPMC, intraductal papillary mucinous carcinoma; MPD, main pancreatic duct**

**Supplementary Table 2. Postoperative outcomes of 94 patients after PSM**

**Group	mBA-PSM (n = 47)	CA-PSM (n = 47)	p**

**C-D ≥ III (case)	12 (25.5%)	24 (51.1%)	0.010**

**POPF (case)	27 (57.4%)	28 (59.6%)	0.834**

**None	20 (42.6%)	19 (40.4%)**

**BL	23 (48.9%)	8 (17.0%)**

**CR	4 (8.5%)	20 (42.6%)	< 0.001**

**PPH grade B/C (case)	1 (2.1%)	9 (19.1%)	0.004**

**Biliary leakage (case)	1 (2.1%)	4 (8.5%)	0.154**

**Drain removal day (day)	7 (4-124)	20 (4-153)	0.124**

**Drain reinsertion (case)	2 (4.3%)	13 (27.7%)	0.002**

**Hospital stays (day)	34 (13-112)	37 (5-230)	0.170**

**Mortality (case)	0 (0.0%)	1 (2.1%)	0.237**

***Values are medians (range)**

**Abbreviations are PSM, propensity score matching; C-D, Clavien-Dindo classification; POPF, postoperative pancreatic fistula; BL, biochemical leak; CR, clinically relevant; PPH, postpancreatectomy hemorrhage**
